# Open the Technical Black Box of Tumor Mutational Burden (TMB): Factors Affecting Harmonization and Standardization of Panel-Based TMB

**DOI:** 10.3390/ijms23095097

**Published:** 2022-05-03

**Authors:** Meng-Ta Sung, Yeh-Han Wang, Chien-Feng Li

**Affiliations:** 1 Division of Hematology and Oncology, Department of Internal Medicine, MacKay Memorial Hospital, Taipei 104217, Taiwan; prozacker@gmail.com; 2 Division of Hematology and Medical Oncology, Mennonite Christian Hospital, Hualien 970472, Taiwan; 3 Division of Pathology and Medical Informatics, ACT Genomics Co., Ltd., Taipei 114065, Taiwan; 4 ACT Precision Medicine Clinic, Taipei 114063, Taiwan; 5 College of Nursing, National Taipei University of Nursing and Health Sciences, Taipei 112303, Taiwan; 6 Institute of Public Health, National Yang Ming Chao Tung University, Taipei 112304, Taiwan; 7 Department of Medical Research, Chi Mei Medical Center, Tainan 710402, Taiwan; angelo.p@yahoo.com.tw; 8 Institute of Precision Medicine, National Sun Yat-Sen University, Kaohsiung 804201, Taiwan; 9 National Institute of Cancer Research, National Health Research Institutes, Tainan 704016, Taiwan

**Keywords:** tumor mutational burden (TMB), next-generation sequencing (NGS), harmonization

## Abstract

As tumor mutational burden (TMB) has been approved as a predictive biomarker for immune checkpoint inhibitors (ICIs), next-generation sequencing (NGS) TMB panels are being increasingly used clinically. However, only a few of them have been validated in clinical trials or authorized by administration. The harmonization and standardization of TMB panels are thus essential for clinical implementation. In this review, preanalytic, sequencing, bioinformatics and interpretative factors are summarized to provide a comprehensive picture of how the different factors affect the estimation of panel-based TMB. Among the factors, poor DNA quality, improper formalin fixation and residual germline variants after filtration may overestimate TMB, while low tumor purity may decrease the sensitivity of the TMB panel. In addition, a small panel size leads to more variability when comparing with true TMB values detected by whole-exome sequencing (WES). A panel covering a genomic region of more than 1Mb is more stable for harmonization and standardization. Because the TMB estimate reflects the sum of effects from multiple factors, deliberation based on laboratory and specimen quality, as well as clinical information, is essential for decision making.

## 1. Introduction

Along with the investigation of immune checkpoint inhibitors (ICIs), tumor mutational burden (TMB) has been developed to be a predictive biomarker in recent years. By definition, TMB refers to the total load of somatic mutations in tumor cells. As somatic mutations may cause specific tumor neoantigens, patients with a high TMB are likely to be responsive to immunotherapy [1,2,3,4]. A high TMB was first noted to be associated with the treatment response of cytotoxic T-lymphocyte-associated protein 4 (CTLA-4) inhibitors in melanoma [5,6]. In the following years, TMB was employed in many clinical trials of anti-programmed cell death protein 1 (PD-1)/programmed cell death protein-ligand 1 (PD-L1) agents to treat various cancer types. Patients with a higher TMB tended to exhibit a better treatment response, but the testing methods and cutoffs of TMB varied across trials [3,7,8,9,10,11,12,13,14].

Several sequencing methods and multi-gene panels have been established to test TMB in academic, medical and diagnostic laboratories. Originally, the gold standard to calculate TMB was whole-exome sequencing (WES), where the total number of somatic mutations was calculated and reported. However, this is less feasible in most clinical settings because of its labor intensiveness and high cost, the lengthy turnaround time and the lack of computational or bioinformatics specialists. Panel-based TMB assays were thus developed by many laboratories and diagnostic companies. Generally, a TMB panel includes several hundreds of genes, and the somatic mutation load in tumors is estimated using specified bioinformatics algorithms. However, as the design varies across panels from gene selection to bioinformatics algorithms, no universal cutoff defines a high TMB status. The variation between TMB estimates can confuse clinicians and may hinder clinical decision making. Further harmonization and standardization are mandatory for the clinical implementation of panel-based TMB assays.

The standard is still unclear and sometimes confusing, although several TMB panels have been approved or cleared by the U.S. Food and Drug Administration (FDA). In the KEYNOTE-158 study, TMB was defined as a predictive biomarker, and a cutoff of 10 mutations per megabase (muts/Mb) to define a high TMB was proposed and further approved by the FDA as a tumor agnostic indication for the prescription of pembrolizumab [9,15]. A commercial laboratory-based panel, FoundationOne (F1) CDx (Foundation Medicine Inc.), was simultaneously approved as the companion diagnostic test for this indication. In addition, the Memorial Sloan Kettering Cancer Center (MSKCC) also developed an in-house cancer genomic profiling assay, MSK-IMPACT (Integrated Mutation Profiling of Actionable Cancer Targets), which was cleared through the FDA 510(k) review in 2017 [16,17]. In the following years, several other next-generation sequencing (NGS) panels also obtained approval from the FDA. However, it is difficult to harmonize the inter-panel variation between NGS panels, regardless of whether they have regulatory authorization or clinical validation data from trials. The present review aims to provide a comprehensive picture regarding the factors that affect the standardization and harmonization between panel-based TMB so that clinicians, pathologists and laboratory scientists can have a better understanding when interpreting TMB results and manage inter-panel variations when making treatment decisions.

## 2. Harmonization and Standardization of Panel-Based TMB

As the harmonization and standardization of TMB are considered to be critical and essential for clinical implementation, international collaboration was initiated in 2018. Two nonprofit organizations—Friends of Cancer Research (FoCR), based in Washington, DC, and Quality Assurance Initiative Pathology (QuIP), based in Berlin, Germany—formed research consortia to involve laboratories and stakeholders from different domains, such as diagnostic companies, academic or medical institutions, pharmaceutical companies and regulatory administrations or government-funded institutions [18]. They planned to execute a series of research projects (FoCR harmonization study) so that a strategy for the harmonization and standardization of panel-based TMB could be proposed. Various factors at different steps of the testing process were investigated in different phases of the FoCR harmonization study (Table 1). The factors involved at different steps that may affect TMB estimates are summarized in Table 2. According to the results, the FoCR harmonization study indicated that panel-based tissue TMB estimates are comparable with WES TMB [19,20].

### 2.1. Preanalytic Factors (Sample and DNA Issues)

Several preanalytic factors may influence the TMB estimation, primarily associated with DNA quality. Poor DNA quality, whether resulting from specimen handling, processing or archiving, is known to cause more false-positive calls of somatic mutations, which often feature a low allele frequency [30]. These false-positive mutations inappropriately increase the TMB estimates. Quy et al. noted that patients with high TMB estimates from specimens with a low DNA library concentration show no to less treatment benefits from anti-PD-L1 antibodies compared to the high concentration group [23]. While a low library concentration reflects poor DNA quality, the high TMB status determined by testing such specimens was more likely to be misclassified. In this study, significant increases in false-positive variant calls were also observed in formalin-fixed paraffin-embedded (FFPE) tissue specimens compared to frozen-fixed fresh tissue, a phenomenon that suggests that formalin potentially damages DNA tissue, especially with improper fixation [22]. Formalin-fixation-induced deamination is one of the preanalytic factors that leads to the overestimation of TMB.

Tumor fraction and input DNA quantity both affect the sensitivity of TMB testing, though their impacts vary in degrees between cancer types and panels. Some cancer types are known to have a higher TMB, such as NSCLC and melanoma, while others mostly express a low TMB. Generally, a low tumor fraction may cause missed calls of tumor somatic mutation and, thus, underestimation of TMB. However, panel-specific bioinformatics algorithms may partially compensate for these effects, and an acceptance criterion of tumor fraction has been verified in some TMB panels. The effects of a low tumor fraction and DNA input can be minimized through routine measures of laboratory quality assurance/quality control (QA/QC).

Although the clinical utility of blood TMB (bTMB) has been increasingly recognized and emphasized, it remains debatable whether bTMB can be harmonized with tissue-based TMB (tTMB) either in its value or clinical interpretation [31,32,33]. On a biological basis, bTMB is dynamic, and its value can be significantly influenced by the amount of circulating tumor DNA (ctDNA) shed from the primary or metastatic tumor to the blood [34,35]. The amount of ctDNA and even the mutation variants can change continuously with the different clinical conditions of a patient, such as the early stage, post-surgical intervention, treatment-naïve advanced disease or under chemotherapy, while the variability in tTMB mostly results from intra- or inter-tumoral heterogeneity [32]. In addition, considering the differences between variants and their profiles detected in tissue and blood samples, the algorithm for variant calling and TMB calculation is designed differently [32,33]. For example, mutations detected from clonal hematopoiesis rather than cdDNA should be further filtered out from bTMB estimation [36]. Therefore, although bTMB may dynamically reflect the response to ICI treatment and prognosis [37,38,39], it is still difficult to harmonize bTMB with tTMB detected by either WES or panels, especially when lacking paired tumor tissue and blood samples obtained simultaneously. Blood TMB is also outside of the scope of the FoCR harmonization study. Further investigation is needed for a better understanding of the potential of bTMB as a predictive biomarker and its correlation with tTMB.

### 2.2. Sequencing Factors

Gene coverage and panel size are key factors affecting the performance of a TMB panel. Genes selected in TMB panels, together with their bioinformatics algorithm, primarily determine the accuracy and variability in TMB estimates [40]. As the genes selected in a panel are mostly cancer-related, their distribution and coverage in the genome are not randomly or evenly distributed. In addition, the prevalence of cancer gene mutations is also different across cancer types. Therefore, gene selection may cause internal bias for panel-based TMB estimates, which requires further calibration by the bioinformatics algorithm. Generally, the combination of gene selection and bioinformatics has been optimized in most laboratories and assays, and it has demonstrated comparability with WES TMB. However, the phase II FoCR harmonization study observed a tendency toward the overestimation of TMB when known pathogenic cancer genes were not excluded from the estimation [20]. Panel size was also critical to determine the variability in a TMB assay. Several studies indicate that smaller panels (<1 Mb) exhibit significant variability when correlating with WES TMB calculation [24,41,42,43]. 

Special considerations regarding the sequencing quality or performance of given genes have been applied when designing and constructing a panel-based TMB. Certain genes or coding regions are excluded from TMB calculation because the sequencing results of such genes are frequently unreliable due to some technical gaps. For example, the sequence of central exons was found to be highly repetitive and variable in some mucin genes, e.g., MUC2 and MUC6 [44]. As the sequence has not been completely resolved, mutation variants are not defined or counted for TMB estimation. 

Sequencing depth is a major difference between panel-based TMB and WES TMB. In comparison with WES, a TMB panel can have increased sequencing depth. More variants with a low allele frequency can be detected than in WES, which should also be taken into consideration when developing a bioinformatics algorithm; otherwise, this could lead to a potential overestimation. In contrast, TMB can be underestimated when using sequencing methods with a low depth, such as whole-genome sequencing, as some somatic mutations may fail to be detected. Special considerations are necessary to construct bioinformatics algorithms so that the TMB estimates can be calibrated if possible. Therefore, sequencing depth is considered an important QC metric. The sensitivity may be reduced if the depth does not meet the QC requirement with proper bioinformatics pipelines deployed [25].

### 2.3. Bioinformatics Factors

The initial clinical investigation of TMB was to calculate the sum of somatic mutations detected by WES. Several calculation strategies based on the inclusion types of mutations have been developed in different studies. Although attempts to include all somatic mutation types (e.g., synonymous, non-synonymous single-nucleotide variations (SNVs) and small insertions and deletions (indel)) for TMB calculations have been reported in several studies [14,45,46,47,48], the calculation that includes missense (non-synonymous) mutations only has been the mainstay approach for WES TMB [26]. Chang et al. investigated the impacts of the two calculation strategies above on the TMB value using CheckMate 026 data and found a perfect correlation between “all mutation types” and “missense mutation only” (Spearman’s rho = 0.99), but the former calculation exhibited 3.1-fold higher TMB values compared to the latter (median TMB: 540 muts for all mutation types while 170 muts for missense mutation only) [26]. As TMB panels only detect hundreds of genes (approximately accounting for 0.5–2 megabase of the genome across panels), various computational approaches have been optimized across laboratories or commercialized panels for a better correlation between panel-based TMB and WES TMB [43]. 

**Figure 1:**
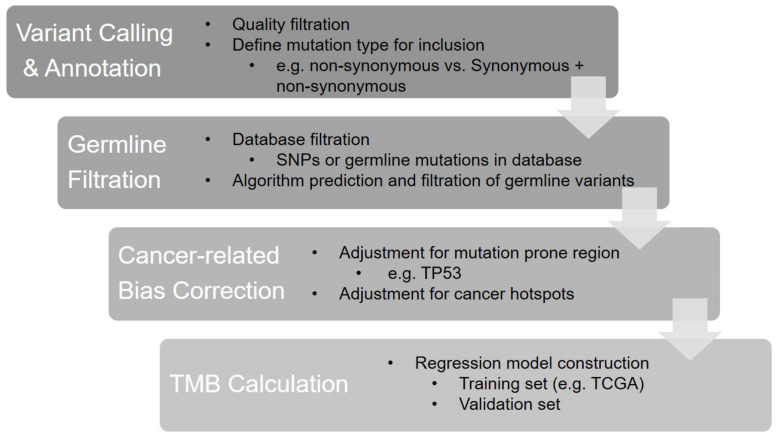
The bioinformatics algorithm of panel-based TMB calculation.

The core steps of the panel-based TMB calculation are shown in Figure 1. First, the step of variant calling and annotation defines true variants based on quality metrics and annotates variant types included for TMB estimation, as well as the genetic information needed for further analysis. In the second step, germline mutations and single-nucleotide polymorphisms (SNPs) are filtered and excluded from TMB calculation. Then, algorithmic adjustment is deployed to correct the bias caused by cancer hotspot mutations, as their high frequency and cancer-type specificity can cause significant deviations in the TMB estimates and lead to a poor correlation with WES TMB. Finally, a regression model is developed and trained by a set of cases with known WES TMB values, and then it is validated using another set.

To achieve a better correlation with WES TMB, many TMB panels have been developed that use bioinformatics approaches that are different from those in WES in terms of variant calling and filtering. For example, in many WES TMB calculations, only non-synonymous mutations are counted. However, both synonymous and non-synonymous mutations are included for bioinformatics computation in some panels, as this approach is believed to increase the sampling of variants in the limited gene list of panels and, thus, is probably better able to represent the overall mutational load across the genome. Although this approach only showed a minimal effect on the correlation between WES TMB based on the FoCR harmonization studies, the data also exhibited reduced inter-panel variability when calculating both synonymous and non-synonymous mutations. The FoCR harmonization study also evaluated the effects of the approach when excluding known somatic pathogenic variants and found that TMB would be overestimated without the filtration of known pathogenic mutation variants [19,20]. The inclusion strategies of mutation types among participating laboratories and panels in the phase II FoCR harmonization study are summarized in Table 2. 

In addition to somatic mutations, germline variant filtering is critical to estimate TMB. Ideally, all germline variants, including both single-nucleotide pleomorphisms (SNPs, ≥1% of population allele frequency) and mutations (<1% population allele frequency), should be excluded from TMB calculation; otherwise, the TMB value will be overestimated. The perfect filtering-out procedure requires a matching normal specimen from the same patient [49]. However, the acquisition of matching normal tissue is not very feasible in clinical scenarios because of the increased cost and potential ethical concerns regarding germline genetic information. Many diagnostic laboratories have developed their TMB panels in the form of tumor tissue specimens only (Table 3). Therefore, optimization of the bioinformatics algorithm is essential to improve the performance of panel-based TMB estimation. 

The population database approach is a standard protocol to filter out germline variants. The most commonly used population databases include GnomAD (the Genome Aggregation Database), TCGA (the Cancer Genome Atlas), ExAc (Exome Aggregation Consortium), 1000 Genomes and ddSNP (the Single-Nucleotide Polymorphism Database) [50]. Some laboratories have also established customized databases to optimize germline variant filtration, as some variant annotations with minor ancestry are not obtained by the public database. Considering the risk of TMB overestimation due to “residual” germline variants, some laboratories have developed in-house filtering algorithms to further identify possible germline variants that are not listed in databases. However, their performance or details are not publicly accessible. Despite these efforts, TMB panels are prone to overestimate TMB in comparison with WES, especially in African American and Asian populations [20,26,49].

### 2.4. Interpretation and Reporting

Cutoff definition is complicated in the clinical application of a TMB panel. In trials using WES TMB, there are various cutoffs ranging from 100 to 248 mutations [3,5,8,51,52,53]. To date, 10 mut/Mb estimated by the F1 CDx assay has been approved by the U.S. FDA as the tumor agnostic high-TMB cutoff, and it is widely used in clinical trials (Table 4). However, different cutoff values provided by F1 CDx or other panels were also investigated in clinical trials and showed statistical significance [4,12,54]. Based on the CheckMate 568 and 026 study, the cutoff of 10 mut/Mb in F1CDx can be bridged to 199 mutations in WES TMB [7,50,55,56]. Although the FoCR harmonization study indicated that the panel-based TMB estimates are comparable with those of WES TMB, the equivalence of 10 mut/Mb in F1 CDx cannot be easily determined in other TMB panels. For example, the cutoff of high TMB for the MSK-IMPACT assay was defined at 13.8 mut/Mb based on their cohort [40]. Further alignment in the numerical cutoff of TMB across panels requires calibration tools and reference datasets or materials [20]. 

In addition, as the TMB level varies in distribution across cancer types [43], whether a universal TMB cutoff works best for every cancer patient is still debatable. Studies have indicated that different cancer types with a similar TMB estimate showed various treatment responses, while the top 20% of TMB values in a given histology of tumors predicted a better treatment response than those with a low TMB [27,28]. However, in some cancer types, microsatellite instability (MSI-H) might be a confounding factor when the clinical utility of TMB is analyzed [57,58]. The expression of other genes or biomarkers may sometimes need to be taken into consideration when interpreting the clinical significance of TMB [43,59]. Other patient factors, including race and treatment history, may also increase the TMB estimate in an individual patient. Therefore, multiple factors complicate the harmonization and standardization of TMB clinical reporting. Special consideration should be emphasized in the report so that clinicians can make decisions on an individual level rather than relying on a universal cutoff. This will be a more “precise” practice in the era of precision medicine.

**Table 4:** Results of important clinical trials that explored high tissue TMB as potential biomarker.

Cancer	Trials/Types	Treatment	Method	TMB Cutoff	RR	PFS	OS
Various cancer types, previously treated	KEYNOTE-158 [9] Single-arm phase II	Pembrolizumab	F1 CDx assay	≧10 mut/Mb	29%	2.1 months	11.7 months
NSCLC	CheckMate227 [7,55,60] Phase III	Nivolumab plus ipilimumab vs. platinum-doublet chemotherapy	F1 CDx assay	≧10 mut/Mb	45.3% vs. 26.9%	7.2 vs. 5.5 months (*p* < 0.001)	NA
NSCLC	Checkmate9LA [61,62] Phase III	Nivolumab plus ipilimumab plus platinum-doublet chemotherapy x 2 cycles vs. platinum-doublet chemotherapy	F1 CDx assay	≧10 mut/Mb	46 vs. 28%	8.9 vs. 4.7 months	mOS:15.0 vs. 10.8 months
NSCLC	Checkmate026 [8] Phase III	Nivolumab vs. platinum-doublet chemotherapy	CGP by research lab	≥243 somatic missense mutations per sample	47 vs. 28%	9.7 vs. 5.8 months	OS: no difference
NSCLC	Checkmate568 [56] Phase II	Nivolumab plus low-dose ipilimumab	F1 CDx assay	≧10 mut/Mb	43.8%	7.1 months	NA
NSCLC	BIRCH [63] Phase II	Atezolizumab	F1 CDx assay	≧10 mut/Mb	25% versus 14%	NA	NA
NSCLC	POPLAR [63] Randomized phase II	atezolizumab versus docetaxel	F1 CDx assay	≧10 mut/Mb	20% versus 4%	7.3 versus 2.8 months	16.2 versus 8.3 months
NSCLC	MYSTIC [64]	Durvalumab versus Durvalumab plus tremelimumab vs. chemotherapy	F1 CDx assay	≧10 mut/Mb	NA	NA	18.6 versus 16.6 versus 11.9 months
UC	IMvigor211 [65]	Atezolizumab versus chemotherapy	F1 CDx assay	>9.65 mut/Mb	NA	NA	11.3 versus 8.3 months HR:0.68 (0.51–0.90)
Melanoma	IMspire170 [66]	Cobimetinib plus atezolizumab versuspembrolizumab	F1 CDx assay	>10 mut/Mb	NA	NR versus 3.7 months in cobimetinib plus atezolizumab arm (*p* = 0.0004) NR versus 3.6 months in pembrolizumab arm (*p* = 0.002)	NA
Melanoma	Checkmate-067 [67]	Nivolumab versus nivolumab plus ipilimumab versus ipilimumab	WES	>median	Nivolumab 62.1% versus 31.5% Nivolumab plus ipilimumab 64.8% versus 51.0% Ipilimumab 25.5% versus 14.3%	HR 0.45 in nivolumab arm; HR 0.55 in nivolumab plus ipilimumab arm; HR 0.60 in ipilimumab arm	HR 0.46 in nivolumab arm; HR 0.53 in nivolumab plus ipilimumab arm; HR 0.52 in ipilimumab arm

## 3. Conclusions

As the clinical utility of TMB as a predictive biomarker for ICIs has been demonstrated in multiple cancer types, the clinical demand for TMB testing has increased in recent years. The standardization and harmonization of various TMB panels are essential for laboratory implementation and clinical interpretation. This article reviewed the technical aspects that have been key to panel harmonization so that we can better understand the complex nature and computation of TMB embedded within the powerful NGS pipeline. Although the FoCR harmonization study has provided experience and principles to establish the possibility of an interchangeable TMB estimate across panels, laboratory measures that assure their testing quality are the real-world foundation to ensure that TMB is successful for clinicians and patients universally. 

## Figures and Tables

**Table 1:** Design and purposes of FoCR harmonization study.

FoCR Study	Design	Purpose
Phase I [19]	In silico analysis using TCGA data	Validate bioinformatics algorithms. Standardize panel-based TMB estimates by comparing reference WES TMB value.
Phase II [20]	Analysis using clinical samples (FFPE tissue)	Evaluate variation between TMB panels.
Phase III *	Retrospective analysis of clinical samples with ICI treatment response	Validate cutoffs of TMB for clinical application.

* No data are published.

**Table 2:** Factors affecting TMB estimates.

Testing Process	Factors Affecting TMB Results	Effects on TMB Estimation
Sample collection and DNA extraction	● Specimen type ■ Blood ■ Plasma ■ Tissue ● Tumor fraction ● Specimen quality and quantity ■ Fixation and storage ■ DNA library concentration	● No available harmonization scheme between specimen types ● Low tumor fraction may lead to underestimation of TMB [21] ● Formalin fixation-induced deamination may cause overestimation of TMB [22] ● Low DNA library concentration may overestimate TMB [23]
Sequencing	● Sequencing gene list ● Panel size ● Sequencing depth	● Small panel size (<1 Mb) leads to greater variability in TMB estimates [24] ● Reduced sequencing depth may lower the sensitivity of the TMB panel [25]
Bioinformatics algorithm	● Variant calling and filtering ■ Somatic variants ■ Germline variants ● Correlation with WES TMB	● No filtration of cancer hotspot mutations causes overestimation of TMB [20] ● Residual germline variants after filtration cause overestimation of TMB [20,26]
Interpretation and reporting	● Cutoff setting ■ Universal cutoffs? ■ Cancer type specific? ■ Adjustment by race? ● Clinical information	● A universal cutoff for high TMB does not predict similar treatment response [27] ● Specific cutoffs for given cancer types may better predict treatment response [28] ● Overestimation of TMB noted in Asian and African American individuals when using certain panels or algorithms [20,26] ● Some anti-cancer drugs, such as temozolomide, or radiation, cause hypermutation of tumor cells and thus increase TMB [29]

**Table 3:** Bioinformatics strategies in TMB panels.

Laboratories/Panels	Mutation Type Included	Known Pathogenic Variant Removal	Germline Variant Removal Approach
ACTOnco+	Non-synonymous + synonymous	Yes	Algorithm-based
AZ650	Non-synonymous + synonymous	No	Matching normal tissue
OncoPanel v3.1	Non-synonymous only	No	Algorithm-based
SureSelectXT	Non-synonymous only	No	Algorithm-based
FoundationOne CDx	Non-synonymous + synonymous	Yes	Algorithm-based
TruSight Oncology (TSO500)	Non-synonymous + synonymous	Yes	Algorithm-based
JHOP2	Non-synonymous + synonymous	Yes	Algorithm-based
MSK-IMPACT	Non-synonymous only	No	Matching normal tissue
NeoTYPE Discovery Profile for Solid Tumors	Non-synonymous + synonymous	No	Algorithm-based
Ion AmpliSeq Comprehensive Cancer Panel	Non-synonymous only	No	Algorithm-based
PGDx elio tissue complete	Non-synonymous + synonymous	Yes	Algorithm-based
QIAseq TMB panel	Non-synonymous only	No	Algorithm-based
Oncomine Comprehensive Assay Plus (OCA Plus)	Non-synonymous only	No	Algorithm-based
Oncomine Tumor Mutation Load Assay (OTMLA)	Non-synonymous only	No	Algorithm-based
